# Fenestrated giant mastoid emissary vein, a novel finding

**DOI:** 10.1007/s00276-026-03869-z

**Published:** 2026-03-27

**Authors:** Mugurel Constantin Rusu, Răzvan Costin Tudose, Alexandra Diana Vrapciu

**Affiliations:** 1 Faculty of Dentistry, Division of Anatomy, “Carol Davila” University of Medicine and Pharmacy Department 1, Bucharest, 050474 Romania; 2 Research Department, “Dr. Carol Davila” Central Military Emergency Hospital, 8 Eroilor Sanitari Blvd, 010825 Bucharest, Romania

**Keywords:** Mastoid emissary vein, Mastoid foramen, Fenestration, Suboccipital venous plexus, Deep cervical vein, Computed tomography, Horos

## Abstract

**Purpose:**

To document an extracranial fenestration of the mastoid emissary vein (MEV) and to clarify its drainage pattern and potential procedural relevance.

**Methods:**

A 46-year-old male underwent multidetector CT angiographic evaluation of the cervical carotid system. DICOM data were post-processed in Horos using multiplanar reconstructions and three-dimensional volume-rendered images; morphometric measurements were obtained on the reconstructions.

**Results:**

A large left MEV (6.6 mm) exited through a single mastoid foramen situated 2.95 cm postero-supero-medially to the mastoid tip. The vein divided 2.2 mm distal to the foramen into an anterior limb (5.0 mm) and a posterior limb (1.1 mm) that rejoined 2.33 cm inferiorly, forming a long fenestration. Three deep communicating veins connected the fenestrated segment to the suboccipital venous plexus, and the distal MEV continued as the deep cervical vein.

**Conclusion:**

Extracranial fenestration is a plausible variant of a prominent MEV. Recognition on CT may prevent misinterpretation as vascular duplication or pathology and may influence mastoid, retrosigmoid, and endovascular planning in the mastoid–suboccipital region.

## Introduction

Emissary veins are vascular conduits that connect intracranial dural venous sinuses with extracranial venous networks via emissary foramina. They are commonly described as lacking valves, which may permit bidirectional blood flow and contribute to pressure equalisation within the venous sinuses [5]. The mastoid emissary vein (MEV) traverses the mastoid emissary canal and mastoid foramen and links the sigmoid sinus with extracranial tributaries, most commonly the posterior auricular and occipital venous systems [7, 13].

Anatomical and imaging studies show that the mastoid emissary canal/foramen complex is frequently present but highly variable, ranging from absence to multiple canals/foramina and broad differences in calibre [5, 6, 14]. In an MDCT series, mastoid emissary foramina were identified in 92.15% of cases, with diameters up to 5.0 mm on the right and 4.4 mm on the left, and with up to six foramina on the left side [5].

The MEV becomes clinically relevant when it is enlarged or serves as a dominant drainage route, as may occur with altered cranial venous outflow. An MEV diameter exceeding 3.5 mm has been considered clinically significant, and injury to a prominent MEV may lead to brisk haemorrhage during posterior mastoid or retrosigmoid approaches [13]. In addition, inadvertent injury may be difficult to control because of bidirectional flow and proximity to the sigmoid sinus [7].

Enlarged MEVs have also been implicated as a venous cause of pulsatile tinnitus and, in symptomatic patients, have been managed by surgical ligation or endovascular occlusion [1, 8, 9, 17]. In otologic implant surgery, prominent MEVs have been directly associated with intraoperative bleeding risk, supporting the value of preoperative imaging review [3].

While previous reports have focused on the number and calibre of mastoid emissary canals/foramina and on enlarged single-channel MEVs, an extracranial fenestration of the MEV has not been emphasised in the morphometric series available to us [5, 6, 13, 14]. We therefore report a fenestrated MEV identified on three-dimensional imaging, with multiple deep anastomoses to the suboccipital venous plexus and continuation as the deep cervical vein, and discuss the anatomical and clinical implications.

## Materials and methods

The archived CTA files of an adult male (46 years) who underwent multidetector computed tomography for routine documentation of carotid anatomy were minutiously observed. Imaging was acquired on a Somatom Definition Edge scanner. The DICOM dataset was imported into Horos for post-processing and visualisation. Multiplanar reconstructions and three-dimensional volume-rendered images were used to assess arterial anatomy and accompanying venous structures in the mastoid–suboccipital region. Morphometric measurements (vein diameters, distances, and lengths) were obtained from the reconstructed images. Measurements were performed using a window width of 229 HU and a window level of 259 HU.

## Results

In an adult male case, a 46-year-old was found to have a peculiar venous variant on the left side during routine documentation of the carotid anatomy.

**Fig. 1:**
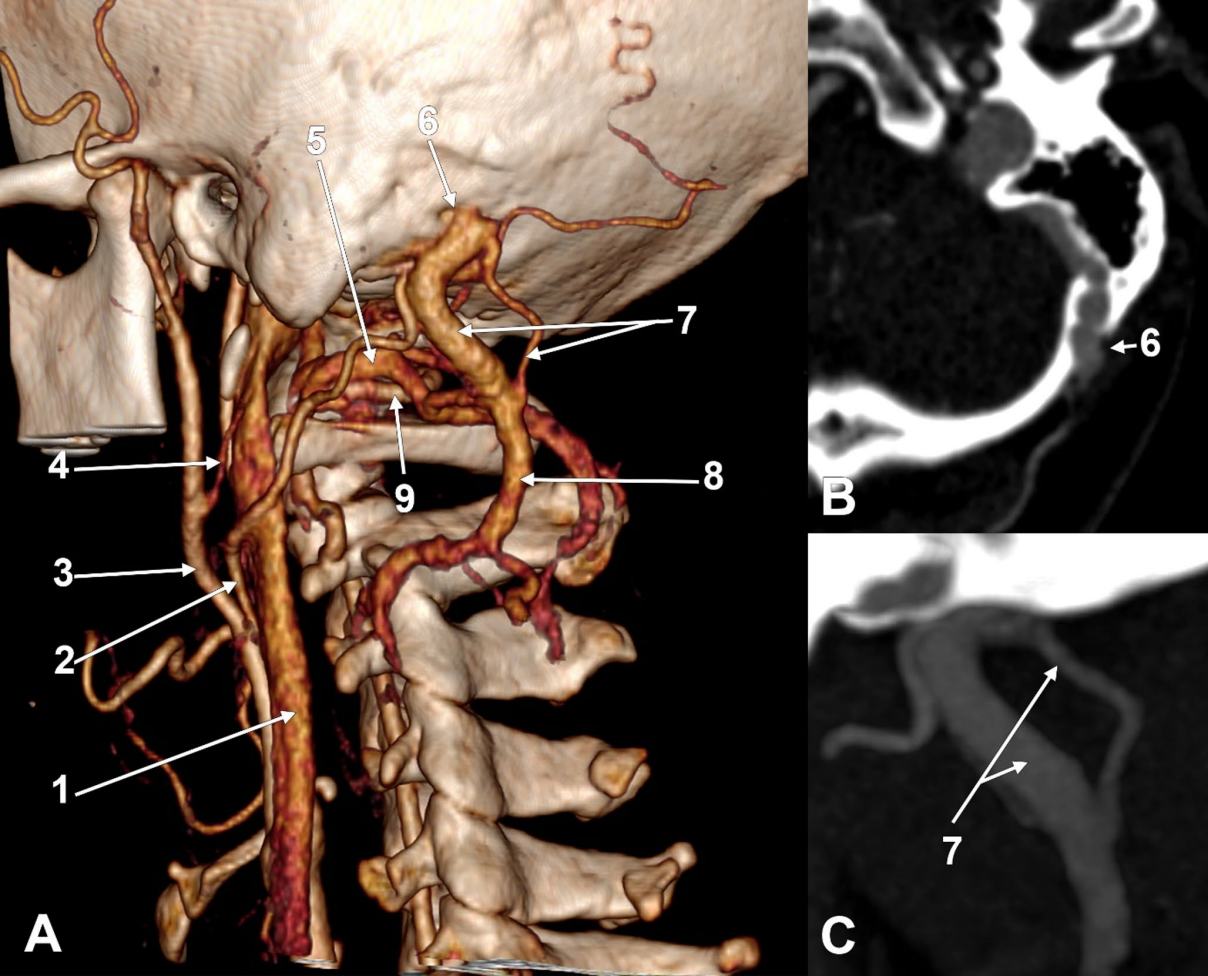
Suboccipital fenestration of the left mastoid emissary vein (MEV). **A** Three-dimensional volume rendering. Left side. Postero-infero-lateral view. **B** Oblique axial slice, inferiorly viewed, demonstrating the unique mastoid foramen. **C** Oblique sagittal slice, laterally viewed, demonstrating the MEV’s fenestration. (1) internal jugular vein; (2) occipital artery; (3) external carotid artery; (4) posterior auricular artery; (5) suboccipital plexus; (6) MEV, mastoid foramen; (7) suboccipital fenestration of the MEV; (8) deep cervical vein; (9) V3 segment of the vertebral artery

The left carotid bifurcation was opposite to the greater hyoid horn. From the external carotid artery (ECA) left, sequentially, the superior thyroid, lingual, occipital (OA), facial, and posterior auricular artery. Then, the ECA terminated postero-medially to the posterior margin of the mandibular ramus, at the midheight of the ramus, with the superficial temporal and maxillary arteries. The OA ascended obliquely deep to the posterior belly of the digastric muscle, crossing the internal jugular vein (IJV), then posterior to the transverse process of the atlas, to reach into the atlantomastoid space. It further crossed the suboccipital venous plexus, consistently supplied by the anterior condylar vein, and reached the cranial OA sulcus. Here, a 6.6 mm large MEV crossed deeply, exiting a single mastoid foramen located at 2.95 cm postero-supero-medially to the mastoid tip (Fig. 1). The mastoid foramen was unique and measured 6.49/6.88 mm in diameter. The sizes of the MEV and mastoid foramen were thus fitted. That MEV was connected to the left sigmoid sinus.

The V3 segment of the right vertebral artery had a 4.1 mm caliber and continued with a 3.3 mm caliber V4 segment. The left vertebral artery had a V3 segment of 2.4 mm, but had a hypoplastic V4 segment of just 1.4 mm. As no pathologic process compressed the left VA at the entrance into the dural sac, we considered that the left VA loss of calibre was determined by the origin of the left posterior inferior cerebellar artery (PICA), of 1.2 mm, in that dural orifice. The right PICA left the V4 segment of the right vertebral artery internally to the jugular tubercle. Distally to the right PICA origin, the V4 segment narrowed at 2.8 mm.

**Fig. 2:**
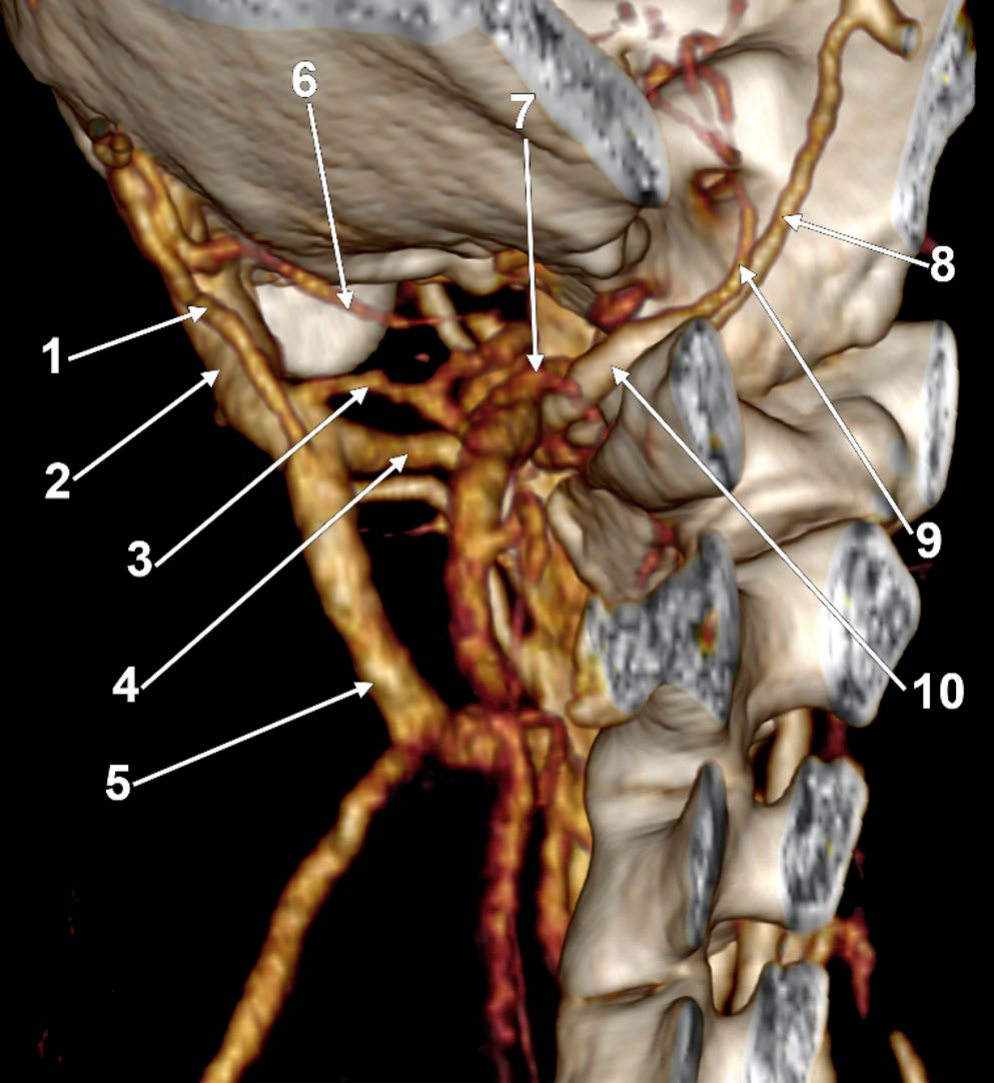
Deep communicating branches of the left mastoid emissary vein (MEV). Three-dimensional volume rendering. Left side. Postero-inferior view. (1) posterior thin arm of the MEV fenestration; (2) anterior large arm of the MEV fenestration; (3) middle communicating vein; (4) inferior communicating vein; (5) deep cervical vein; (6) superior communicating vein; (7) suboccipital venous plexus; (8) V4 segment of the vertebral artery; (9) posterior inferior cerebellar artery; (10) V3 segment of the vertebral artery

The left MEV (Figs. 1 and 2) was divided at 2.2 mm distal to the mastoid foramen into a 5.0 mm antero-lateral arm and a 1.1 mm postero-medial one that reunited at 2.33 cm inferiorly, thus forming a fenestration of the MEV. Both arms of that fenestration were crossed medially by the OA.

The MEV had three deep anastomoses with the suboccipital plexus, all connected to the larger arm of the MAV’s fenestration: an upper one connected to the large anterior arm of the fenestration at 6.2 mm distal to the mastoid foramen, a middle one at 5.4 mm above the inferior end of the fenestration, and an inferior one at 2.7 mm above that end. Distal to the fenestration, the MEV continued as the deep cervical vein.

## Discussion

Emissary veins emerge during fetal development as auxiliary outflow channels when the rapidly enlarging transverse sinuses outpace the capacity of the sigmoid sinus and IJV, and a variable number persist postnatally with highly diverse morphologies [12]. The extracranial fenestration documented here most likely reflects incomplete coalescence of parallel plexiform venous channels during this developmental process, representing part of the recognised spectrum of extraaxial developmental venous anomalies [10].

The present case demonstrated a large MEV (6.6 mm) exiting a single mastoid foramen and forming a 2.33 cm-long extracranial fenestration shortly distal to the foramen, with three deep communicating veins to the suboccipital venous plexus and distal continuation as the deep cervical vein.

From a morphometric perspective, the diameter observed here is well above the typical ranges reported in imaging and dry skull series. There were reported mastoid emissary foramen diameters up to 5.0 mm on the right and 4.4 mm on the left in an MDCT cohort [5]. D’Mello et al. (2025) summarised that most MEVs in published series are < 2–3 mm, with enlarged veins (> 4–5 mm) forming a small minority [3]. Our 6.6 mm MEV exceeds the 3.5 mm threshold proposed as clinically significant and falls within the range that has been associated with operative bleeding in modern otologic series [3, 13].

Multiplicity of the bony pathway, multiple mastoid emissary canals/foramina or accessory canals, has been described in anatomical and imaging studies, including high-resolution CT and cone-beam CT [5, 6, 11, 13, 14]. In our case, however, the bony exit was single, and the variation occurred extracranially as a split-and-rejoin segment (fenestration). Such a configuration could be misinterpreted as two independent veins on non-contrast imaging and may be relevant if the MEV is evaluated as a surgical corridor landmark or as a target for occlusion.

The three anastomoses with the suboccipital venous plexus and the continuation as the deep cervical vein support the concept that the MEV can participate in posterior neck venous drainage pathways. Emissary veins may assume a major collateral outflow function when normal jugular outflow is altered [13], and the multiple extracranial communications in our case could represent a robust collateral route. The MEV was recently highlighted as one of the named tributaries of the condylar emissary venous plexus in the occipitoatlantal region, further underscoring its role in the suboccipital venous communication network and its potential to contribute to uncontrolled intraoperative bleeding during upper cervical procedures [16].

Unilateral vertebral artery hypoplasia is not an uncommon finding, with a pooled prevalence of 13.41% reported in a recent meta-analysis of 176,391 subjects across 32 studies encompassing the V3 and V4 segments [15], and prevalences as high as 40.9% recorded in large MRI series when a broader diameter threshold is applied [2]. The calibre reduction of the left V4 in our case is therefore consistent with a recognised anatomical variant rather than acquired stenosis, further contextualised by the fact that a transdural origin of the PICA, itself a facultative content of the VA dural ring, was identified in 5.56% of vertebral arteries in a dedicated CTA prevalence study [4].

Venous pulsatile tinnitus due to enlarged MEV has been documented as unilateral or bilateral and has been managed successfully by surgical ligation or endovascular coiling/embolization in selected patients [1, 8, 9, 17]. Although the present case was identified during anatomical documentation rather than in a symptomatic setting, a fenestrated segment may be clinically relevant if occlusion is planned, because persistent flow through an unoccluded limb could theoretically maintain symptoms or promote collateralisation.

From a surgical perspective, the mastoid and suboccipital regions are frequently traversed in posterior fossa surgery and are increasingly traversed in otologic implant procedures. High-resolution CT has been recommended for identifying mastoid emissary anatomy preoperatively [6, 13], and D’Mello et al. (2025) emphasised a review of prior temporal bone CT to mitigate avoidable bleeding risk during Osia implantation [3]. Our findings extend this message by showing that clinically relevant variation may occur beyond the bony canal, emphasising careful evaluation of the extracranial venous segment when contrast-enhanced imaging is available.

Limitations of this report include its single-case design and the lack of haemodynamic or clinical correlation. Nevertheless, recognition of extracranial fenestration as a possible MEV variant adds nuance to radiological interpretation and may assist surgeons and interventionalists working in the posterior mastoid and upper cervical region.

## Data Availability

The dataset used and analyzed during the current study is available from the corresponding author upon reasonable request.
